# The risk factors for tuberculosis in liver or kidney transplant recipients

**DOI:** 10.1186/1471-2334-14-387

**Published:** 2014-07-11

**Authors:** Jia Liu, Jin Yan, Qiquan Wan, Qifa Ye, Yisheng Huang

**Affiliations:** 1 Nursing School of Central South University, Changsha 410013, China; 2 Department of Transplant Surgery, The Third Xiangya Hospital, Central South University, Changsha 410013, China; 3 Nursing Department, the Third Xiangya Hospital, Central South University, Changsha 410013, China; 4 Department of Internal Medicine, Thoracic Hospital of Hunan Province, Changsha 410013, China

**Keywords:** Tuberculosis, Infection, Transplantation, Risk factor

## Abstract

**Background:**

Liver or kidney transplant recipients are at a higher risk of developing tuberculosis (TB) than general population. We aimed to clarify the incidence density of and risk factors for TB in liver or kidney transplant recipients in the present study.

**Methods:**

All patients with TB following liver or kidney transplantation were investigated retrospectively at the Third Xiangya Hospital, Central South University, Changsha, China. The incidence density of TB was calculated. We performed a nested case–control study (1:1) to investigate by univariate and multivariate logistic regression analysis the potential risk factors for TB.

**Results:**

From January 2000 to August 2013, 1748 kidney and 166 liver transplant recipients were performed at a university teaching hospital. Among the 1914 recipients, 45 cases (2.4%) of TB were reported. The incidence density was 506 cases per 10^5^ patient-years in kidney or liver transplant recipients, which was 7 times higher than in the general Chinese population (around 70 cases per 10^5^ person-years). The median time to develop TB was 20.0 months (interquartile ratio: 5.0-70.0). The receipt of a graft from a cadaveric donor (odds ratio [OR] = 3.7; 95% confidence interval [CI] = 1.4-10.0; *P* = 0.010) and the preoperative evidence of latent TB (OR = 6.8; 95% CI = 2.0-22.7; *P* = 0.002) were identified as two risk factors for developing TB in liver or kidney transplant recipients.

**Conclusions:**

The incidence density of TB among liver or kidney transplant recipients was much higher than in the general Chinese population. Recipients receiving a graft from a cadaveric donor and the preoperative evidence of latent TB were two major risk factors for developing TB in liver or kidney transplant recipients.

## Background

Tuberculosis (TB) remains a major global health problem. The incidence rate of TB among transplant recipients relies on its incidence rate in the general population. Recipients following organ transplantation are placed at a 20-74 times higher risk for developing TB than the general population [1-5]. TB in solid organ transplant (SOT) recipients is a challenge because of its atypical and extrapulmonary presentations, metabolic interactions between the immunosuppressive drugs and the drugs used to treat TB, the side effects from long-term treatment of TB, as well as a high mortality rate [1,6-9]. Among transplant recipients, the development of TB is mainly caused by reactivation of an old dormant infection [10-12]. China is one of the world’s 22 countries with the highest burden of TB and has great liver and kidney transplant activity. We aimed to clarify the incidence density of and risk factors for TB in liver or kidney transplant recipients in China in the present study.

## Methods

### Ethics statement

The study protocol, which included participants providing written consent prior to the study, was approved by the Third Xiangya Hospital, Central South University, Medical Ethical Committee.

### Patient population and definitions

From January 2000 to August 2013, all liver or kidney transplant recipients with TB were identified using the electronic medical record system at the Third Xiangya Hospital, Central South University, Changsha, China; their clinical and demographic characteristics were carefully reviewed. A nested case-control study was then performed to reveal the association between risk factors and TB following liver or kidney transplantation.

A patient was considered to have TB if *Mycobacterium tuberculosis* was isolated by culture, acid-fast bacilli were present on the smear, polymerase chain reaction was positive for *Mycobacterium tuberculosis*, or caseating granulomas were found in histopathology [13,14]. Patients whose TB was diagnosed and treated on the basis of clinical or radiological suspicion were excluded from the study. Old TB lesions on chest x-ray were defined as fibrotic pulmonary lesions in upper lobes or anywhere in the lung. Incidence density was calculated by the number of new cases divided by the population-time (person-years of observation) in which they occur. Controls were randomly chosen at a ratio of 1:1 from recipients who had liver or kidney transplantation not complicated by TB at the same time as the cases. Subjects in the control group were well matched to the cases with regard to gender, transplant organ type, and date of transplantation (±1 year). Clinical records of liver or kidney recipients with TB were analyzed including demographic characteristics, diabetes mellitus, etiology of renal or hepatic insufficiency, chronic hepatitis B or C virus infection, previous transplantation, pure protein derivative (PPD) skin test, transplant organ type, graft origin, immunosuppressive regimen, rejection episode within 6 months prior to TB, cytomegalovirus (CMV) infection and major infections within 3 months prior to TB, TB sites, diagnostic methods, time of TB onset, and body temperature at the onset of TB. CMV infection was defined as biopsy-proven CMV disease or reactivation with positive PCR detection of CMV DNA/positive antigen detection of PP65. Patients who entered the cohort most recently were followed up for at least 3 months after transplantation.

### Statistical analysis

Results for continuous variables with a normal distribution were presented as mean ± standard deviation (SD) and compared using Student’s *t*-test. Chi-square analysis or Fisher’s exact test was used to compare categorical data. Discrete variables were expressed as percentages. Factors associated with TB on a univariate analysis with *P* < 0.05 were introduced into a multivariate logistic regression analysis. Odds ratio (OR) with 95% confidence interval (CI) was calculated to assess the association of potential risk factors with the development of TB. A two-tailed value of *P* < 0.05 was established as the threshold of statistical significance. Data analyses were performed with the statistical package SPSS, version 17.0 (SPSS Inc., Chicago, Illinois, USA).

## Results

From January 2000 to August 2013, a total of 1914 kidney and liver transplants were performed at the Third Xiangya Hospital (1748 kidney and 166 liver transplants). Patients were followed for a median 6.2 years post-transplant (interquartile range 2.6-11.8), for a total 725 person-years of follow up. During this time 45 patients developed active TB, corresponding to an incidence density of 506 per 10^5^ person-years. Among these 45 cases with TB, there were 34 men and 11 women; 43 received kidney transplants and 2 received liver transplants. The mean age of TB patients at transplantation was 37.9 (37.9 ± 10.0) years. Pulmonary TB was the most common form of the disease and was diagnosed in 28 patients (62.2%). The disease was disseminated in 13 (28.9%) patients. Extrapulmonary TB occurred in four (8.9%) patients (TB lymphadenitis, TB peritonitis, TB spondylitis, and tuberculocele in one patient each). The median interval from the date of transplantation to the development of TB was 20.0 months (interquartile ratio: 5.0-70.0). Of the TB cases, 17 (37.8%) appeared within the first posttransplant year. Of the 45 cases, all underwent preoperative chest x-ray, 36 PPD test, and 4 interferon gamma release assay (IGRA) test. Seventeen patients showed evidence of latent TB. Old TB lesions on chest x-ray existed in 10 patients, the PPD test result was positive for 10 patients, and IGRA test was positive for 1 patient (Table 1). Of the 45 controls, all underwent preoperative chest x-ray, 16 PPD test, and 1 IGRA test. Five showed evidence of latent TB. Screening of latent TB was, but treatment of latent TB was not, the standard of care in our setting. Thus, none of cases and controls with the present of evidence of latent TB received prophylaxis with isoniazid prior to, or in fact after transplantation. *Mycobacterium tuberculosis* cultures were available in 38 out of 45 cases (84.4%). Of these 38 cases, 23 had a positive culture (60.5%). Of the 45 patients with TB, 4 patients died, and of these deaths, 2 (50.0%) were due to disseminated TB.

**Table 1:** Main characteristics of 45 kidney or liver transplant patients with TB

**Characteristics**	**No. (%) (n = 45)**
Age, mean years ± SD	37.9 ± 10.0
Gender, number of male/number of female	34/11
Temperature of 39°C or greater	15 (33.3)
Primary kidney or liver disease, n (%)	
Glomerulonephritis	33 (73.3)
Diabetes	7 (15.6)
Adult polycystic disease	1 (2.2)
Liver cirrhosis	2 (4.4)
Other	2 (4.4)
Donor type, n (%)	
Cadaveric	32 (71.1)
Living	12 (26.7)
DCD	1 (2.2)
The type of transplantation, n (%)	
Liver	2 (4.4)
Kidney	43(95.6)
Two or more transplants	4 (8.9)
Immunosuppressive drugs, n (%)	
Methylprednisolone	45 (100)
Prednisone	45 (100)
Mycophenolate mofetil	44 (97.8)
Tacrolimus	34 (75.6)
Cyclosporine	10 (22.2)
Sirolimus	1 (2.2)
Antilymphocytic or antithymocytic agents	7 (15.6)
Time of TB diagnosis posttransplant, no. of cases (%)	
< 7 mo	13 (28.9)
7th to 12th mo	4 (8.9)
> 12 mo	28 (62.2)
Evidence of latent TB (No. of positive test/No. of patients tested)	17 (37.8)
Untreated TB lesion in chest plain radiograph	10/40 (25.0)
Preop PPD test positive	10/36 (27.8)
Preop IGRA test positive	1/4 (25.0)
Recent TB contact history	1/45 (2.2)
Type of TB, n (%)	
Pulmonary	28 (62.2)
Extrapulmonary^1^	4 (8.9)
Disseminated	13 (28.9)
Diagnostic modalities (No. of positive test/No.of patients tested)	
Culture positive	23/38 (60.5)
Smear positive	15/36 (41.7)
PCR positive	12/20 (60.0)
Pathology only	3/3 (100.0)
IGRA positive	6/30 (20.0)
PPD positive	5/20 (25.0)
Diabetes mellitus, n (%)	7 (15.6)
Rejection within 6 months prior to TB, n (%)	15 (33.3)
CMV infection within 3 months prior to TB	18 (40)
HCV, HBV	10 (22.2)
Major infection within 3 months prior to TB	13 (28.9)
ESR at the onset of TB > 40 mm/h	18 (40.0)
Creatinine at the onset of TB > 2 mg/dl	10 (22.2)
Crude mortality	4 (8.9)

^1^Cases with extrapulmonary TB in 4 patients, TB lymphadenitis, TB peritonitis, TB spondylitis, and tuberculocele in 1 patient each.

TB, tuberculosis; SD, standard deviation; DCD, donation after cardiac death; PPD, pure protein derivative; IGRA, interferon gamma release assay; PCR, polymerase chain reaction; CMV, cytomegalovirus; HCV, Hepatitis C Virus; HBV, Hepatitis B Virus; ESR, Erythrocyte Sedimentation Rate.

Eighteen cases (40.0%) had CMV infection within 3 months prior to TB presentation. All 7 cases with a history of diabetes mellitus (15.6%) underwent a kidney transplant. Six cases had hepatitis B and 4 hepatitis C virus infection prior to transplantation. The general characteristics of these 45 kidney or liver transplant patients with TB were described in Table 1.

Table 2 described the clinical characteristics of kidney or liver transplant recipients with TB compared with controls using the univariate and multivariate analysis. The univariate analysis showed that CMV infection within 3 months prior to TB (*P* = 0.012), the receipt of a graft from a cadaveric donor (*P* = 0.031) and the preoperative evidence of latent TB (*P* = 0.003) were more frequent in the case group, but age, gender, immunosuppressive drugs received, diabetes mellitus, history of rejection or use of antilymphocytic or antithymocytic agents prior to TB, hepatitis B or C virus infection, and the occurrence of major infections prior to diagnosis of TB did not differ between cases and controls (Table 2). The potential risk factors that were consistently retained in the multiple logistic regression analysis were the receipt of a graft from a cadaveric donor (OR = 3.7; 95% CI = 1.4-10.0; *P* = 0.010) and the preoperative evidence of latent TB (OR = 6.8; 95% CI = 2.0-22.7; *P* = 0.002).

**Table 2:** Clinical characteristics of kidney or liver transplant recipients with TB compared with controls

**Characteristics**	**Cases (n = 45)**	**Controls (n = 45)**	***P***
Univariate analysis			
Age, mean years ± SD	37.9 ± 10.0	37.8 ± 10.0	0.974
Gender, number of male/number of female	34/11	34/11	-
Donor type, Deceased/the others	32/13	22/23	0.031
The type of transplantation, Liver/Kidney	2/43	2/43	-
Two or more transplants	4	4	1.000
Tacrolimus/Cyclosporine	34/10	34/11	0.849
Use of antilymphocytic agents	7	14	0.081
Preoperative evidence of latent TB^1^	17	5	0.003
Diabetes mellitus	7	7	1.000
Rejection, n (%)	15	13	0.649
CMV infection	18	5	0.012
HCV, HBV	10	10	1.000
Major infection^2^	13	16	0.499
Multivariate analysis	OR	(95% CI)	
Graft from a cadaveric donor	3.7	(1.4-10.0)	0.010
Preoperative evidence of latent TB	6.8	(2.0 - 22.7)	0.002

^1^Including positive PPD or IGRA, untreated TB lesion on plain chest radiograph, recent TB contact history.

^2^Including pneumonia,bloodstream infections,urinary tract infection or intracranial infection.

TB, tuberculosis; SD, standard deviation; DCD, donation after cardiac death; CMV, cytomegalovirus; HCV, Hepatitis C Virus; HBV, Hepatitis B Virus; ESR, Erythrocyte Sedimentation Rate; OR, odds ratio; CI, confidence interval.

## Discussion

TB is one of the most important opportunistic infections encountered posttransplantation [9,11,15,16]. The incidence rate of TB in SOT recipients ranged from 1.2% to 15% [1]. According to the 2013 WHO global TB report, China ranks as the second among the world’s 22 high burden countries with a TB incidence around 1.4 million, and the incidence density of TB in general Chinese population was around 70 cases per 10^5^ person-years in 2012 [17]. The incidence density of TB in our present study was 506 per 10^5^ patient-years, indicating that a liver or kidney transplant recipient had 7-fold higher risk of developing TB than a person from the general Chinese population.

Previous studies [9,11,13] had demonstrated that the majority of SOT patients developed TB within 1 year of operation. In the present study, we found that less than half (37.8%) of all TB cases appeared within the first posttransplant year. The possible explanation for this result was that in the current study, the subjects with TB mainly comprised kidney transplant recipients (43 out of 45 TB cases). Kidney transplant recipients were less immunosuppressed and lived longer than other transplant recipients, which could have accounted for the later occurrence of TB in these population [9].

Pre-transplantation parameters that could predict the development of TB would be extremely valuable since the classic presentation of TB was atypical among transplant recipients. Various variables previously described in the literature as risk factors for TB include: age, racial background, blood group, diabetes mellitus, previous exposure to TB, protein-calorie malnutrition, hemodialysis for longer periods, chronic liver disease (in kidney transplant), type of transplantation and immunosuppressive agents, allograft rejection, chronic graft dysfunction, hepatitis C virus or CMV infection, and concomitant opportunistic infections [9,11,14,15,18-27].

There was an understandably high rate of pre-existing positive PPD and chest x-ray changes in our study population (37.8% in cases vs. 11.1% in controls), accordant with Basiri A and colleagues [14] who reported that 64.2% of patients and 0.2% of controls had radiological evidence compatible with tuberculosis. In view of this, we would expect high rates of transmission of TB after renal or liver transplantation. Actually, an incidence 2.2% (1/45) of donor-derived transmission of TB was found in the current study, consistent with other studies [9,28] which suggested that in American, donor-derived transmission of TB accounted for < 5% of TB cases after SOT. We found in the present study that the preoperative evidence of latent TB was an independent risk factor for posttransplant TB, similar to other studies [14,18], but having major discrepancy from some reports [25,29] that did not confirm the association between latent TB and risk of TB. Although some transplant centers have observed a low likelihood of the development of TB in untreated PPD-positive transplant recipients without other risk factors for TB [3,30-32], several existing guidelines made recommendations that all patients awaiting organ transplant should be screened for latent TB infection, and that patients diagnosed with latent TB infection should be ideally treated pretransplant or if time does not permit, therapy should be started or completed posttransplant [19,33-35]. Our finding highlighted the importance of treatment of latent TB infection, which was recommended by other studies [11,29,36,37].

Another finding of ours was that the receipt of a graft from a cadaveric donor was independently associated with an increased risk for developing TB following liver or kidney transplantation. The reasons for this result were not clear. One possible explanation was that the remainder of the donors mainly comprised living relative donors (12 out of 13 non-cadaveric donors). Protective immunity against TB infection was a cell-mediated process [38,39]. The ability of T-cell to inhibit the growth of *mycobacteria* was impaired more in patients receiving a graft from a cadaveric donor than in patients receiving a graft from a living relative donor, because more immunosuppressive agents must be administrated against acute cellular rejection. There were many other factors contributing to predisposing SOT recipients to TB, such as higher rates of cadaveric donor active or latent TB infection leading to a certain incidence rate of donor-derived transmission of TB [9,28]. Thus, patients receiving a graft from a cadaveric donor might be at a higher risk of TB. Our study contributed to the literature by demonstrating, for the first time, that the receipt of a graft from a cadaveric donor could be a risk factor for increased TB among liver or kidney recipients. The results of this study might be useful in avoiding TB in liver or kidney transplant recipients.

In short, we found the incidence density of TB to be 7-fold higher among liver or kidney transplant recipients than among the general Chinese population. The receipt of a graft from a cadaveric donor rather than other sources of donors, and the preoperative evidence of latent TB were significant risk factors for developing TB in liver or kidney transplant recipients. Nevertheless, the small sample size and the retrospective nature of the study design were two major limitations in the present study. Clinicians should pay more attention to these risk factors and properly administer preventive therapy to improve the outcome of liver or kidney transplant recipients.

## Conclusions

The incidence density of TB among liver or kidney transplant recipients was much higher than in the general Chinese population. Recipients receiving a graft from a cadaveric donor and the preoperative evidence of latent TB were two major risk factors for developing TB in liver or kidney transplant recipients.

## Competing interests

The authors declare that they have no competing interests.

## Authors’ contributions

Concept/design and critical revision of article were done by professor QY and JY. Data collection was done by JL and YH. Analysis and drafting article were finished by Doctor QW. All authors read and approved the final manuscript.

## Pre-publication history

The pre-publication history for this paper can be accessed here:

http://www.biomedcentral.com/1471-2334/14/387/prepub
